# The March towards a Vaccine for Congenital CMV: Rationale and Models

**DOI:** 10.1371/journal.ppat.1005355

**Published:** 2016-02-11

**Authors:** Kristy M. Bialas, Sallie R. Permar

**Affiliations:** 1 Human Vaccine Institute, Duke University Medical School, Durham, North Carolina, United States of America; 2 Department of Pediatrics, Duke University Medical School, Durham, North Carolina, United States of America; University of Michigan Medical School, UNITED STATES

## What Is the Global Impact of Congenital Cytomegalovirus (CMV) Infection?

CMV is a ubiquitous human herpesvirus that causes a lifelong, persistent infection in its host. Whereas primary CMV infections in otherwise healthy individuals are typically asymptomatic and go unnoticed, complications can develop in immunosuppressed individuals following acute CMV infection or CMV reactivation, presenting as retinitis, hepatitis, pneumonitis, gastroenteritis, or other end-organ diseases [1]. Similar severe and life-threatening conditions can arise following CMV infection of the developing fetus, resulting in lasting neurologic deficits, and while not as well known as other common causes of pediatric disabilities, congenital CMV (cCMV) is the leading infectious cause of hearing loss and cognitive impairment in newborns worldwide [2].

The impact of cCMV infection on pediatric health is significant, affecting 0.5%–2% of all live-born infants worldwide [1]. In the United States, approximately 30,000 congenital infections occur annually, of which more than than 5,000 infections lead to permanent disabilities, including sensorineural hearing loss (SNHL), growth restriction, seizures, and motor and cognitive disability [1,2]. An estimated 10% of infected infants exhibit neurological sequelae at birth, while an additional 10%–15% of infected infants develop SNHL in the first two years of life, making CMV the leading nongenetic cause of childhood SNHL [1,2]. Much like the rubella vaccine effectively eliminated congenital rubella syndrome in the Americas, a maternal vaccine that elicits protective immune responses is needed to eliminate CMV as a major cause of birth defects.

## What Is Known about Immune Factors That Protect against Congenital CMV?

Development of a protective maternal CMV vaccine will require a complete understanding of the immunological factors that prevent cCMV transmission. However, this is challenging because even natural CMV infection, which elicits the most robust and prolonged adaptive immune response of any other human pathogen, does not protect against superinfection or eliminate placental virus transmission. In fact, cCMV transmission occurs at a rate of 1%–2% in seropositive women, accounting for at least two-thirds of all congenital infections [1]. CMV immunity prior to conception does, however, appear to provide at least partial protection to the fetus, as evident by the significantly higher rate of cCMV transmission (40%) among seronegative women who newly acquire CMV during pregnancy [1]. In addition, congenitally infected infants born to CMV-seropositive women are less likely to have clinical sequelae at birth and rarely develop neurological deficits other than SNHL [1]. Efforts to determine these naturally protective immune factors have identified three maternal immune responses potentially associated with reduced risk of transmission: CMV-specific CD4+ T cell responses, CMV-specific antibody avidity, and neutralizing antibodies directed against the recently identified CMV glycoprotein complex gH/gL/UL128-UL130-UL131, which is required for CMV entry into epithelial cells [3–5]. Thus, to date, many of the human vaccine trials have focused on the induction of avid and potent neutralizing antibodies rather than cell-mediated responses.

## How Have CMV Vaccine Candidates Performed in Clinical Trials?

Live-attenuated CMV vaccines were the first vaccine platform to be evaluated in humans. While proving to be safe, tolerable, and immunogenic in seronegative subjects, live-attenuated CMV vaccines have not been successful at boosting immune responses in seropositive individuals and have failed to prevent acquisition of primary CMV infection among seronegative women exposed to young children actively shedding CMV [1]. Furthermore, although live-attenuated CMV vaccination also failed to protect against primary CMV infection in seronegative, immunosuppressed renal transplant recipients, vaccination of this cohort has been shown to consistently reduce the occurrence of severe CMV disease by more than 85% [1]. Recent efforts to make live-virus vaccines more effective have included recombination with less-attenuated CMV strains and co-administration with interleukin 12 [1]. Though recombinant CMV strains were unsuccessful in enhancing vaccine-elicited immune responses compared to wild type, the addition of interleukin-12 increased both the magnitude and duration of CMV-specific antibodies and T cell response in seronegative subjects. Given the moderate success of live-attenuated vaccines in preventing disease in transplant recipients and the potential to improve immune responses when combined with other vaccine strategies, future studies should continue to explore this platform for the prevention of CMV infection.

Subunit vaccination has been another approach taken to prevent CMV infection. The majority of subunit vaccines have incorporated the CMV surface glycoprotein B (gB). CMV *gB* mediates entry into all cell types and is a dominant target of the CMV-specific humoral response. The most studied gB subunit vaccine is recombinant protein gB adjuvanted with the squalene-based adjuvant MF59, which proved to be safe and immunogenic in Phase I studies in healthy seronegative and seropositive adults, transplant recipients, and young children [6]. In a Phase II study, three doses of gB/MF9 was shown to be 50% efficacious at preventing infection in CMV-seronegative young mothers over a 42-month timespan. The study suggests that a strong CMV vaccine candidate should indeed elicit a robust gB response but will likely need to include additional immunogens such as the newly described gH/gL/UL128-UL130-UL131 pentamer, since this protein complex is an important target of the neutralizing antibody response [6]. Subunit vaccines which utilize viral vectors have also shown promise in humans. Among them is the canarypox vector expressing gB (ALVAC-CMV), which by itself is poorly immunogenic in seronegative subjects but when used as a prime for live-attenuated strains elicits neutralizing antibody titers that match those found in naturally immune sera [7]. Similar canarypox vaccines expressing CMV pp65, a major T cell antigen, are under assessment for their ability to induce cell-mediated immunity, since a vaccine which elicits both arms of the adaptive immune response may necessary for complete protection [8]. Addressing this hypothesis, a Phase III trial assessing the efficacy of a bivalent CMV DNA vaccine (ASP0113), encoding both gB and pp65, to prevent mortality in seropositive transplant recipients is ongoing [9] and, in Phase II studies, elicited robust, pp65-specific T cell responses and late, gB-specific B cell responses.

The induction of high-avidity, highly neutralizing, CMV-specific maternal antibodies is believed to play an important role in the prevention of cCMV transmission and protection of the infant from severe sequelae [4,5]. In support of this hypothesis, administration of hyperimmune globulin to pregnant women with primary CMV infection was shown to reduce the rate of congenital CMV transmission and disease in a nonrandomized study [10]. However, these results were confounded by a follow-up Phase II randomized, controlled trial, in which delivery of hyperimmune globulin during the first 26 weeks of pregnancy in acutely infected women revealed no significant difference in the rate of cCMV transmission between treated and untreated women [11]. The failure of this trial to prevent cCMV transmission may have in part been due to the small treatment group sizes, the inability to control for the timing of CMV infection, the use of suboptimal immunization schedules, or the lack of selection for CMV-specific antibodies with high neutralizing potency. Future studies that select strongly neutralizing monoclonal antibodies specific for the key CMV surface glycoproteins that prove to be most critical for placental virus transmission, as well as studies that evaluate pharmacokinetics to optimize vaccine regimens, could improve future attempts to prevent cCMV transmission by passive infusion.

## What Role Do Animal Models Play in Understanding Protection against cCMV Transmission?

The species-specificity characteristic of CMVs makes preclinical testing of human CMV vaccine candidates in animal models a challenge for predicting clinical outcome. Still, animal models remain valuable tools for the assessment of immune correlates of protection against CMV and the safety and immunogenicity of novel vaccine strategies. Of the small animal models commonly used for preclinical testing, the guinea pig is unique in that its species-specific CMV can cross the placenta and establish fetal infection [1], and thus it has been used to evaluate many of the vaccine strategies that have undergone trials in humans (Table 1) [12–18]. Each vaccine approach in guinea pigs elicited favorable immune responses that mimicked those of wild-type CMV infection and resulted in reduced rates of cCMV infection when administered to pregnant females. Of note, a recombinant gB subunit vaccine, which showed moderate success at preventing primary CMV infection in humans, was effective at preventing congenital CMV transmission in the guinea pig model. However, the anatomical differences between humans and small animal models limit their value in assessing potential vaccine efficacy, as demonstrated by the success of passive antibody infusion in preventing fetal infection in guinea pigs but not in controlled human trials.

**Table 1:** Results of CMV vaccination in the guinea pig model of cCMV and women of child-bearing age.

Vaccine Platform	Efficacy in the guinea pig model of cCMV	Vaccine-elicited responses and protection in women of child-bearing age
**Live-attenuated Vaccines [1,12]**	*Reduced the rate of congenital infection (of live pups) by up to 51% [18]	Elicited short-lived, humanCMV-neutralizing antibody responses in healthy, seronegative women (peak geometric mean titer = 349)
	Reduced the rate of pup mortality by up to 55%	Did not protect against primary HCMV infection of women with infants actively shedding HCMV
**Recombinant Protein Vaccines (gB) [1,7]**	Reduced the rate of congenital infection (of live pups) by up to 36%	Elicited HCMV-neutralizing antibody responses in seronegative adults when given in three doses with MF59 adjuvant, which exceeded the levels found in naturally seroimmune individuals (peak geometric mean titer = 14,098)
	Reduced the rate of pup mortality by up to 62%	Proved to be 50% efficacious at preventing primary HCMV infection in women of child-bearing age
**Viral Vector Vaccines (Alphavirus) [13,14]**	Studies conducted using replicon expressing HCMV pp65 homolog guinea pig CMV (GPCMV) UL83	Studies conducted using replicon expressing HCMV gB or pp65/IE1 fusion protein
	Reduced the rate of congenital infection by 38%	Elicited HCMV-neutralizing antibodies (peak GMT = 218) and functional, HCMV-specific T cell responses (peak mean SFC = 138 gB; 504 pp65; 113 IE1) in healthy, seronegative adults
	Reduced the rate of pup mortality by 44%	
**CMV DNA Vaccines [1,15,16]**	Studies conducted using GPCMV gB or HCMV pp65 homolog GPCMV UL83	Administration of bivalent HCMV gB/pp65 vaccine alone to HCMV-seronegative subjects resulted in modest, gB-specific antibody responses in 22% of vaccinees and T cell responses in 29% and 38% for gB and pp65, respectively
	Reduced the rate of congenital infection by 9% and 36% for gB and UL83 vaccines, respectively	Vaccination of HCMV-seronegative adults with a trivalent HCMV gB/pp65/IE1 vaccine effectively primed memory T cell responses to live-attenuated vaccines
	Neither gB or UL83 vaccine had an effect on the rate of pup mortality	
**Antibody Infusion [11,17,18]**	Studies used hyperimmune anti-gB serum or anti-gH/gL monoclonal antibody	HCMV hyperimmune globulin was administered to women who acquired primary HCMV infection between five and 26 weeks of gestation every four weeks
	Infusion with anti-gB serum reduced the rate of congenital infection by up to 39%	Treatment with HCMV hyperimmune globulin reduced the rate of congenital CMV transmission by 14% (95% CI = -3, 31; *p* = 0.13)
	Infusion with anti-gB serum and gH/gL monolconal antibody reduced the rate of pup mortality by 100% and 35%, respectively	

*Reduced rates of congenital infection and pup mortality in guinea pigs was calculated by subtracting the rates observed in treated dams from untreated dams.

**Fig 1:**
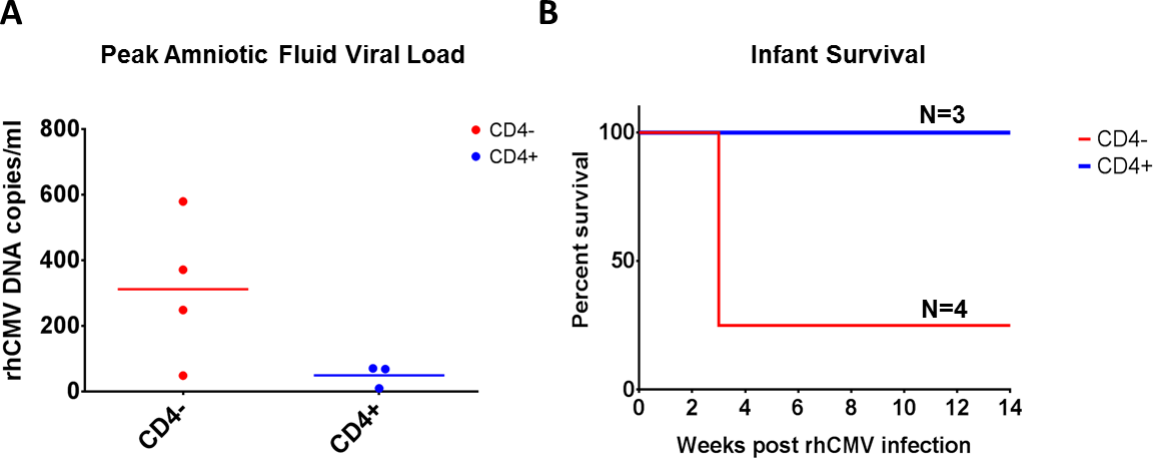
Virus transmission and pregnancy outcome in a novel nonhuman primate model of congenital rhCMV infection. (A) The rate of congenital rhCMV transmission in CD4 T cell depleted (red, CD4-) and nondepleted (blue, CD4+) dams following primary maternal rhCMV infection was determined by detection of rhCMV DNA in amniotic fluid (AF) using quantitative PCR. Peak AF viral loads and mean copy number for CD4- and CD4+ females are shown. (B) Percent survival of fetuses following maternal rhCMV inoculation.

Recently, there has been a push towards the development of a more relevant large-animal model to study cCMV transmission. Rhesus monkeys are widely used for preclinical testing of human vaccines and have an extensive tool set available to assess vaccine-elicited immune responses. Rhesus CMV (rhCMV) is the most characterized nonhuman primate CMV and is more genetically similar to human CMV than rodent CMVs. In fact, vaccine studies to prevent CMV acquisition in these animals are already underway. Recent work in our laboratory demonstrated the capacity of rhCMV to cross the placenta when administered intravenously to seronegative pregnant females early in pregnancy (Fig 1) [19]. Additionally, in support of earlier studies conducted by Lilleri et al., we observed more severe fetal outcome in dams lacking CD4+ T cell immunity who also demonstrated delayed rhCMV-neutralizing antibody responses [4,6]. Moreover, Barry and colleagues showed that direct inoculation of the fetal brain with rhCMV induces similar neurological defects to what is seen in congenitally infected human infants [20]. While establishment of a rhesus model of cCMV transmission will be beneficial for analysis of vaccine efficacy prior to designing clinical trials that will require large cohorts of women to establish efficacy in prevention of cCMV, small animal models will remain useful for early-phase preclinical studies because they are economically more feasible and more high-throughput.

## What Are the Future Challenges for cCMV Vaccine Design?

As development of a CMV vaccine progresses, several important challenges need to be addressed. First, it remains necessary to further define the natural immune correlates of protection against cCMV infection, including the contribution of maternal antibody responses and cell-mediated immunity. Secondly, defining the CMV immunogens which elicit the most robust protective immune responses and block infection of both fibroblast and epithelial cell types is critical for eliminating all cCMV infections. Finally, it is imperative to raise public awareness about the impact of cCMV so that when a successful vaccine becomes available, society will advocate for this novel immune intervention to prevent birth defects and brain damage among infants.
